# The Effects of Physical Exercise on Adolescents’ School Adjustment and Path Analysis—Evidence from the China Education Panel Survey

**DOI:** 10.3390/bs15121602

**Published:** 2025-11-21

**Authors:** Shaohua Tang, Hanwen Chen, Tianci Lu, Baole Tao, Jun Yan

**Affiliations:** College of Physical Education, Yangzhou University, Yangzhou 225127, China; mz120210767@stu.yzu.edu.cn (S.T.); dx120230091@stu.yzu.edu.cn (H.C.); dx120240094@stu.yzu.edu.cn (T.L.); dx120190064@yzu.edu.cn (B.T.)

**Keywords:** physical exercise, adolescents, school adjustment, China Education Panel Survey

## Abstract

Considering the role of school adjustment in the development of adolescent populations, it is particularly essential to find ways to improve the school adjustment of middle school students and to develop further the socialization of adolescents. Based on the 2014–2015 data of the China Education Panel Survey, the effects of physical exercise on adolescents’ school adjustment and possible mechanisms were systematically explored. Based on the ordinary least squares method, instrumental variable method, and propensity score matching method, the results of the study showed that physical exercise significantly improved adolescents’ school adjustment. The results of the mediation effects test indicated that physical exercise not only enhances adolescents’ school adjustment by alleviating negative emotions, but also enhances adolescents’ school adjustment by improving prosocial behavior. This study reveals the mechanism of physical exercise and adolescents’ school adjustment, and provides reasonable and effective suggestions for better promoting adolescents’ school adjustment and formulating related policies.

## 1. Introduction

School serves as a primary microsystem for adolescent development, where effective adjustment behaviors signal strong social adaptation and psychological well-being (León Moreno et al., 2021). School adjustment encompasses adolescents’ comfort in school, task engagement, and academic performance (Demirtaş & Ergene, 2019). It fosters academic success, socio-emotional competence, and life satisfaction (Tomás et al., 2020; Mella et al., 2021). Therefore, for adolescents, adjustment to school life is a crucial early developmental task and one of the most important indicators of their overall health and development (Hou et al., 2022). However, previous studies have found that school adjustment declines during adolescence (Azpiazu et al., 2024), including in China, where 18–35% of adolescents face maladjustment (Wu et al., 2024). Adolescents’ school adjustment is influenced by many factors such as the family environment, the school environment, and the psychological development within the individual (C. Zhang & Gai, 2011). Therefore, identifying interventions to enhance adjustment and exploring their mechanisms holds significant theoretical and practical value.

### 1.1. The Relationship Between Physical Exercise and School Adjustment

In recent years, many countries have elevated physical activity guidelines for children and adolescents to national strategies, positioning physical exercise as an effective tool for adolescent development (Guo & Rao, 2021). However, research on physical exercise’s role in promoting adolescents’ school adjustment yields inconsistent results. Some studies indicate that physical exercise facilitates interpersonal interactions, emotional sharing, and broader social networks (Z. Chen & Yu, 2015), enabling adolescents to manage school changes and academic pressures, thus enhancing adjustment (Cao et al., 2023). Our previous cross-sectional study through Havighurst’s Integrated Developmental Task Theory of Adaptation Development also confirmed that physical activity enables high school students to accomplish their current stage of developmental tasks in the school environment, positively predicting their school adjustment (H. Chen et al., 2024). Havighurst’s Developmental Task Theory of Integral Adaptation suggests that “developmental tasks” are specific things, jobs, or tasks that need to be accomplished at various stages of an individual’s growth (Seiffge-Krenke & Gelhaar, 2008). Individuals who are able to successfully complete developmental tasks at each stage are considered healthy or well-adjusted individuals. Conversely, individuals who fail to achieve developmental tasks may experience negative emotions and may even fail in the socialization process.

Conversely, other work from our team found physical exercise positively associated with adjustment but not a significant predictor (Yan et al., 2020), while another study showed moderate-intensity exercise’s effects fully mediated by intervening variables (Cao et al., 2018). The latter finding, in particular, highlights that the effect of exercise may not be direct but rather operate through underlying mechanisms. The mixed results call for further investigation that can account for such complexities. Therefore, this study aims to re-examine this relationship while controlling for potential confounders and exploring possible mediators. Based on this, we hypothesize H1: Physical exercise promotes adolescents’ school adjustment.

### 1.2. The Mediating Role of Negative Emotions

The present study concluded that physical exercise can improve adolescents’ school adjustment by ameliorating negative emotions. Negative emotion is a generalization of an individual’s subjective experience of grief and displeasure, including a variety of distasteful emotional states such as anger, contempt, disgust, guilt, fear, sadness, and tension (S. Li, 2023). Developmental Task Theory recognizes that emotional development is one of the primary factors influencing critical periods in life, and adolescence is one of those critical periods (Havighurst, 1975). The theory emphasizes the role of positive emotional experiences after the completion of a “developmental task” in facilitating the achievement of subsequent tasks. Negative emotions, on the other hand, can lead to maladaptation by narrowing an individual’s thinking and action resources, creating a tendency to take particular actions (e.g., avoidance), and reducing an individual’s ability to know and do (Fredrickson, 2001). Students high in negative affect are prone to peer–teacher difficulties and classroom disengagement (Hernández et al., 2018) and, during adolescence, show strong links with anger, anxiety, depression, and externalizing behaviors (Y. Zhang, 2023), all of which undermine school adjustment and socialization.

The amelioration of negative emotions is inextricably linked to physical exercise, which reduces anxiety and depression and mitigates stress-related negative affect (Singh et al., 2023). Meta-analytic evidence indicates protective effects across region, age, and gender (Schuch et al., 2018). Physiological accounts attribute these benefits to increased expression of brain-derived neurotrophic factor (BDNF), modulation of the hypothalamic–pituitary–adrenal axis, and reduced systemic inflammation (Lu et al., 2023), mechanisms that alleviate depressed mood and enhance emotional stability. Exercise also augments positive affect via endorphin release, social connection, goal attainment, and body-image gains (Yang et al., 2023). Within the Broaden-and-Build framework, such positive emotions can “undo” the lingering effects of negative states and restore adaptive functioning (Fredrickson, 2001). Positive emotions generated through physical exercise may then counteract the negative effects previously experienced by the individual due to negative emotions, thus restoring a good state. Based on the above theoretical analysis and rationale, this study proposes the hypothesis H2: Negative emotions play a mediating role in the process of physical exercise to promote adolescents’ school adjustment.

### 1.3. The Mediating Role of Pro-Social Behavior

In addition to the mechanisms described above, physical exercise can promote adolescent school adjustment by fostering prosocial behavior. Prosocial behavior, defined as voluntary actions that benefit others without obvious self-gain (e.g., helping, sharing, and comforting), is a core indicator of positive socialization (Eisenberg et al., 2015). Developmental task theory underscores the importance of this mechanism, positing that the development of socially responsible behavior constitutes one of the principal developmental tasks of adolescence (Havighurst, 1975). Successful achievement of this task is fundamental to school adjustment, as evidence indicates that adolescents higher in prosocial behavior report more positive attitudes toward school, stronger classroom relationships, greater participation in collaborative learning, and fewer problem behaviors (Collie et al., 2018; Saleem et al., 2021). Social learning theory provides a clear mechanism for how physical exercise cultivates such prosocial behavior. This theory suggests that individuals develop prosocial behaviors by witnessing the behaviors and outcomes of others during physical exercise, and through learning and reinforcing these behavioral outcomes (Duan et al., 2022). Physical exercise may therefore be a potentially effective measure that can develop prosocial behavior in adolescents (J. Li & Shao, 2022). For example, during physical exercise, adolescents can learn to handle competition and cooperation rationally and acquire prosocial qualities such as humility, respect, solidarity, and helping others (Wan et al., 2021). In summary, developing prosocial behaviors helps adolescents better adapt to life while protecting them from negative developmental outcomes. Based on the above theoretical analysis and rationale, this study proposes Hypothesis H3: Prosocial Behavior mediates the process of physical exercise in promoting adolescents’ school adjustment.

### 1.4. Current Study Aims

To meet the urgent need for scalable, school-embedded levers that enhance adolescents’ school adjustment, we examine physical exercise for three reasons. First, it is a modifiable behavior routinely delivered in schools and aligned with national youth physical-activity strategies, making it readily actionable for education systems. Second, theory links physical exercise to negative emotions and prosocial development providing clear, testable mechanisms. Third, the empirical record is mixed, with many studies relying on cross-sectional designs, small samples, and limited control for endogeneity, which obscures causal interpretation (Bai et al., 2022; Wu et al., 2024; Mosoi et al., 2020). Clarifying whether and how exercise relates to school adjustment therefore has direct implications for both education and public-health policy.

Our study advances this literature in three ways. First, we use the nationally representative 2014–2015 China Education Panel Survey (CEPS), addressing concerns about small, nonrepresentative samples. Second, beyond baseline regression, we implement instrumental-variables estimation and propensity score matching to mitigate confounding and strengthen causal claims. Third, we probe mechanisms by testing whether negative emotions and prosocial behavior mediate the exercise–adjustment association. Together, these contributions extend the evidence base on determinants of school adjustment and inform feasible, school-based strategies to support adolescents’ successful socialization.

## 2. Methods

### 2.1. Participants

This study is based on the analysis of data from the China Education Panel Survey designed and implemented by the China Center for Survey and Data at Renmin University of China. The data samples were selected from 112 schools in China using various sampling methods such as hierarchical, multi-stage, probability proportional to size (PPS), and other sampling methods, and the students, parents, teachers, and school administrators in the seventh and ninth grades were surveyed, which is highly representative of the whole country. The CEPS database not only comprehensively collects all the demographic information of the students, but also includes the data of their corresponding families and schools, which can better meet the needs of this study. Given the research topic and the need for timeliness of the data, this study used the most recent 2014–2015 CEPS follow-up survey data from this database, which contained a total of 9920 samples. After removing samples containing key missing values, 8707 valid samples were retained, the age of the samples was 14.52 ± 0.69 years.

### 2.2. Variable Selection

#### 2.2.1. School Adjustment

The dependent variable of this study was school adjustment. The indicators correspond to B2, B3, B4, and B6 of the academic growth section of the CEPS student questionnaire, and this study classified adolescent school adjustment into three dimensions divided into academic adjustment, interpersonal adjustment, and behavioral adjustment. Among them, academic adjustment mainly refers to students’ adjustment to learning activities. Given that the CEPS data asked three questions about the respondent adolescents’ receptivity to the core curriculum (math, Chinese course, and English), this study used learning ability as a proxy variable for examining academic adjustment, with the question “Do you feel overwhelmed by learning math (language, English) now?”, and the options were all 4-point fixed-order measures. Regarding interpersonal and behavioral adjustment, the options were all 4-point ordinal measures. Interpersonal adjustment includes students’ perceptions of good class and school climate, and three questions were selected: “Most of the students in my class are friendly to me, my class has a good classroom culture, and I feel close to the people in this school.” Behavioral adjustment includes the extent to which students comply with behavioral norms in the classroom and at school, and three questions were selected: “I often participate in activities organized by the school or class, I am bored at this school, and I wish to go to another school”. In this study, the three dimensions of school adjustment were combined to form a composite score for subsequent analysis, with higher scores indicating greater school adjustment among adolescents.

#### 2.2.2. Physical Exercise

The independent variable in this study was physical exercise, and the CEPS data included a survey on the physical exercise status of the interviewed students, which was used to measure the degree of students’ participation in physical exercise through the question “How many days per week and how many minutes per day”. Referring to previous studies (Hu & Yu, 2019), this study first excluded the sample extremes that answered “more than 360 min of exercise per day”, and then calculated the average daily exercise time according to the formula (average daily exercise time = weekly exercise days × daily exercise time/7). In order to make the dependent variable more normally distributed, and so that cases with an average daily exercise time of 0 (no exercise) are not excluded from the sample, this paper adds 0.01 to the value of each case before taking the natural logarithm of the “average daily exercise time”. A normally distributed continuous variable is constructed.

#### 2.2.3. Negative Emotions

The indicators corresponded to C25 of the physical and mental health section of the CEPS student questionnaire, and according to the practice of existing research (W. Li & Lu, 2023), the nine questions of “In the past seven days, have you been frustrated; depressed unable to concentrate on things; unhappy; life is not interesting; unable to lift up the energy to do things; sad, upset; nervous; worrying excessively; foreboding that something bad is happening” were used as indicators of adolescents’ negative emotions. The options were all five-point ordinal measures. The data were processed by summing the results of the nine question scores, with higher scores indicating higher levels of negative emotions felt by the adolescents.

#### 2.2.4. Prosocial Behavior

The indicators corresponded to D1 of the physical and mental health section of the CEPS student questionnaire, and based on existing practices (Wan et al., 2021), three questions were selected as indicators of adolescents’ pro-social behaviors: “Helping the elderly to do things”, “Being in order and queuing up consciously”, and “Treating others with sincerity and friendliness”. Three questions were selected as indicators of adolescents’ prosocial behavior. The options were measured on a five-point ordinal scale. For data processing, the scores of the three questions were summed up, and higher scores indicated higher levels of prosocial behavior among the adolescents.

#### 2.2.5. Control Variable

There are various factors affecting adolescents’ school adjustment, including the family environment, the school environment, interpersonal factors, intra-individual psychosocial developmental factors, as well as social and environmental factors resulting from social changes (Hillekens et al., 2023). In this study, control variables were categorized into three types: individual characteristics, family characteristics, and school characteristics. The control variables in terms of individual level include gender, only child, own health status, health education status, obesity perception and live in school. The family environment is one of the key factors in the positive development of adolescents, in which the level of education of the parents, the relationship with their children, and the economic status of the family may affect adolescents’ physical activity participation and school adjustment. Therefore, the control variables at the family level in this study included students’ household economic status, both parents’ educational attainment, father–child relationship, mother–child relationship, and parental relationship. In addition, the nature of the school, its ranking, its location, and the construction of school physical exercise facilities may also affect physical exercise and school adjustment; for this reason, the control variables at the school level of this study include the type of school, the ranking of the school, the location of the school, and the school physical facilities of the students. Table 1 shows the results of descriptive statistics of the main variables of this study.

### 2.3. Empirical Strategy

Using Stata 16.0, we implement a four-step analytical strategy: ordinary least squares (OLS) regression, an instrumental-variables (IV) approach, propensity score matching (PSM), and Karlson–Holm–Breen (KHB) mediation analysis.

#### 2.3.1. OLS Regression

To estimate the effect of participation in physical exercise on adolescents’ school adjustment, we specify the following linear model:

$${\mathrm{School}\;\mathrm{Adjustment}}_{i}\;=\;\alpha \;+\;\beta_{1}{\mathrm{Physical}\;\mathrm{Exercise}}_{i}\;+\;\beta_{2}{\mathrm{Control}}_{i}\;+{\;µ}_{i}$$

#### 2.3.2. Instrumental Variables (IV) Estimation

OLS estimates may be affected by endogeneity, preventing clear causal interpretation. Two concerns are salient: first, potential bidirectional causality (school adjustment may influence exercise behavior); second, unobserved confounding, even after an extensive set of controls. If such unobservables are correlated with both exercise and school adjustment, OLS coefficients will be biased. To address this, we use the instrumental variable for students’ physical exercise time (excluding the individual) as an instrumental variable and conduct robustness checks within a two-stage least squares (2SLS) framework.

#### 2.3.3. Propensity Score Matching (PSM)

Due to the limitations of observational (nonexperimental) data, adolescents’ decisions to engage in physical exercise are nonrandom, being shaped by observable characteristics such as age, gender, and BMI, as well as household economic status, cultural capital, and parental education. Consequently, OLS regressions are susceptible to selection bias in two respects: self-selection into exercise driven by the aforementioned individual and family observables; and counterfactual misspecification—when OLS uses the school-adjustment outcomes of non-exercising adolescents as the counterfactual for exercising adolescents, pre-treatment differences between treated and control groups undermine identification. To strengthen inference on the relationship between adolescents’ exercise participation and school adjustment, we employ the PSM method of Rosenbaum and Rubin to correct for estimation bias arising from observable heterogeneity (Rosenbaum & Rubin, 1985):

$$\mathrm{ATT}\;=\;E\{E\left[{\mathrm{School}\;\mathrm{Adjustment}}_{1i}|D_{i}\;=\;1,p(X)\right]\;-\;E[\mathrm{School}\;\mathrm{Adjustmen}t_{0i}|D_{i}\;=\;0,p(x)]\}$$

#### 2.3.4. KHB Mediation Analysis

To examine the mediating mechanisms through which physical exercise affects adolescents’ school adjustment, we apply the Karlson–Holm–Breen (KHB) method (Kohler et al., 2011). KHB offers two advantages: broad applicability to mediation analysis under linear (OLS) models, and direct decomposition of the total effect of the key explanatory variable into indirect effects operating through specified mediators (e.g., negative emotions, prosocial behavior) and the remaining component. This enables a transparent assessment of the relative contribution of each mediating pathway.

## 3. Results

### 3.1. Regression Modeling of Physical Exercise as It Affects Adolescents’ School Adjustment

To investigate whether physical exercise has an effect on adolescents’ school adjustment, the present study conducted regression analyses using average daily exercise time as the treatment variable and total school adjustment score as the dependent variable. Since school adjustment is a continuous variable, it was estimated using the OLS model. However, the CEPS collects a large number of samples, and there may be measurement errors or heteroskedasticity due to other reasons, so direct OLS estimation may bias the results. In view of this, the White test for heteroskedasticity was conducted first, and the result showed that *p* < 0.01 indicated the existence of heteroskedasticity, so the regression analysis was conducted using “OLS + Robust Standard Error”. In this study, the control variables of individual characteristics, family characteristics and school characteristics were included in the process of regression results in order to explore more information, and the results are shown in Table 2, in which only physical exercise was included in Column (1), and the results showed that physical exercise significantly and positively affected the school adjustment of adolescents. The results in Columns (2) through (4) show that physical exercise increased its explanatory power on school adjustment from 4.3% to 25.7% after controlling for the control variables. The control variables related to individual and family characteristics are all significantly different, while some of the control variables at the school level are not significantly different, indicating that the variables related to individual and family characteristics are the main factors affecting adolescents’ school adjustment. At this point, physical exercise still significantly affects adolescent school adjustment and remains sufficiently robust, which suggests that physical exercise can effectively contribute to the level of adolescent school adjustment, and thus Hypothesis 1 of this study is preliminarily validated.

### 3.2. Robustness Tests

#### 3.2.1. Instrumental Variable Method

This study was validated using the Durbin–Wu–Hausman Test (DWH), which provides robust results even in the presence of heteroscedasticity. To conduct the DWH test, it is necessary to select an appropriate instrumental variable, and in this study, based on the relevant literature and with reference to previous studies, the school-level physical exercise participation rate (in addition to the individual) was finally selected as the instrumental variable for students’ physical exercise time (Liu et al., 2021; Y. Zhang, 2023). The reasons for choosing this instrumental variable are as follows: first, the overall physical activity participation rate of a school reflects not only the importance attached to the promotion of students’ participation in physical exercise at the school level, but also the degree of improvement of the school’s physical facilities, which has a direct impact on students’ physical exercise. Second, there is no evidence to suggest that physical exercise participation at the school level directly affects adolescents’ level of school adjustment. Therefore, all things considered, this study hypothesizes that school-level physical exercise participation rate excluding individual factors is a reasonable and valid instrumental variable.

Table 3 demonstrates the results of the test using the 2SLS method. The strength of the instrumental variable also affects the results of the test; according to the data in Table 3, the F-values of the first stage in columns (1) to (4) are well above the empirical standardized value of 10, which shows that there is a strong correlation between school-level physical exercise participation (except for individuals) and the endogenous variables, indicating that the instrumental variable is a strong instrumental variable (Stock et al., 2002). In addition, the DWH test results showed *p* < 0.001, indicating the endogeneity of the regression model in this study and the correctness of using the instrumental variable method. The results of the first stage indicate that after the gradual addition of relevant control variables, school-level physical participation (except for individuals) still has a significant positive effect on physical exercise, confirming the rationality of the choice of instrumental variables in this study. The results of the second stage suggest that physical exercise can positively influence adolescents’ school adjustment. The regression results of the instrumental variables were consistent with the OLS regression results, further validating and enhancing the robustness of the OLS regression results.

#### 3.2.2. Propensity Score Matching

**Figure 1:**
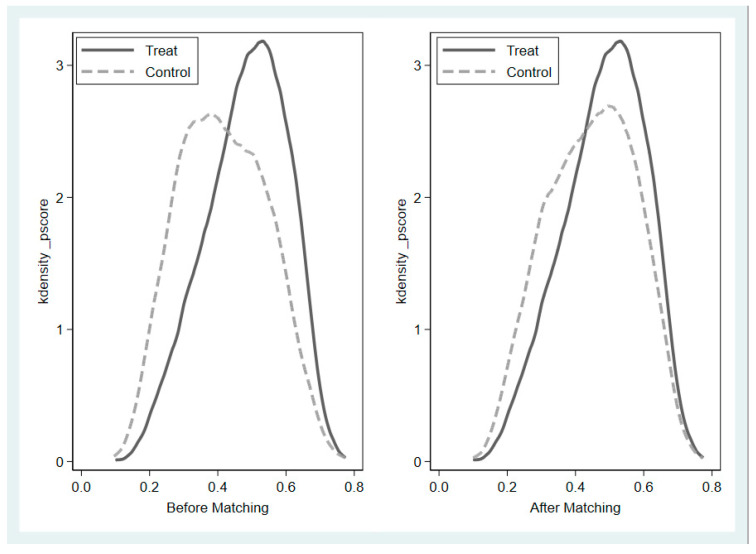
Kernel Density Distribution Before and After Matching.

Given the presence of many other variables confounding the relationship between physical exercise and school adjustment, this may cause sample selection bias, making it difficult to obtain inferences on the net and causal effects of the intervening factors, which in turn affects the accuracy of the results. Therefore, PSM was used in this study to correct the OLS regression results. Adolescents who exercised ≥3 times per week for ≥30 min per session were scored as 1 (treatment group), and those who did not meet this criterion were scored as 0 (control group) to fulfill the prerequisites for the application of PSM. Second, to increase the reliability of the results, this study used kernel density function plots to portray the balance of covariates before and after matching (see Figure 1).

**Figure 2:**
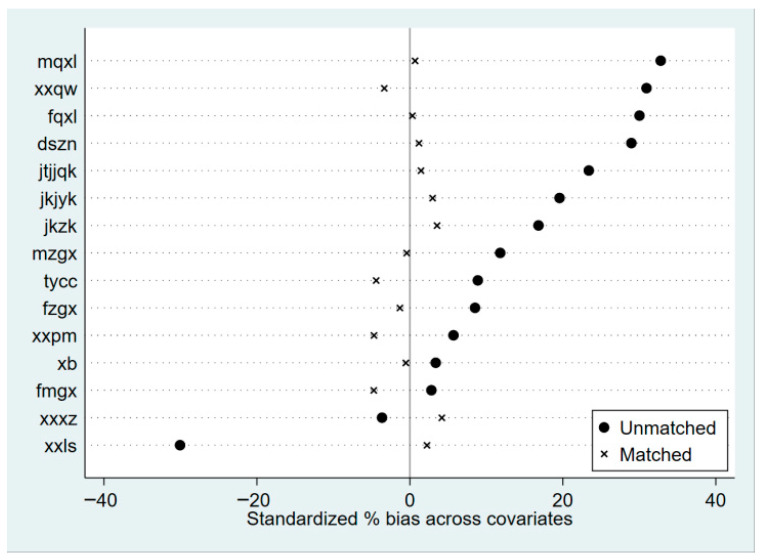
Control Variable Distribution Test Results.

According to Figure 1, the peak of the distribution of school adjustment in the treatment group before matching was shifted more to the right compared to the control group, indicating that the school adjustment of adolescents who regularly participated in physical exercise was superior to that of adolescents who did not regularly participate in physical exercise. The gap between the kernel density curves of the treatment and control groups was significantly narrowed after matching, and the trend was more consistent, while this feature facilitated the comparison of the treatment effect after matching (ATT). In addition, this study further evaluated the balance of the data after matching by calculating the double t-distribution test for individual covariates, and the results, as shown in Figure 2, showed that the standardized deviations of all variables after matching were no more than 4.5% and none of the p-values were significant, which fulfilled the conventional requirement that the absolute value of the standardized deviation was less than 20%, indicating that there was no significant difference between the treatment group and the control group. This indicates that the PSM effectively reduces the differences in explanatory variables between the treatment and control groups, which helps to reduce the impact of sample selection bias on the accuracy of estimation results.

On the basis of the covariate balance test, in order to accurately assess the average treatment effect of physical exercise on adolescents’ school adjustment, three commonly used matching methods were used for estimation in this study: nearest-neighbor matching (k = 4), radius matching (r = 0.03), and kernel matching (using the default kernel function and bandwidth). The results in Table 4 indicate that all three matching methods show that physical exercise significantly improves adolescents’ school adjustment, with effect sizes ranging from 1.037 to 1.055. Compared to the OLS regression results, the effect of physical exercise on adolescents’ school adjustment derived from the PSM was more significant, suggesting that adolescents who participated in physical exercise regularly performed better on school adjustment compared to those who exercised less, controlling for other similar factors. Therefore, Hypothesis 1 of this study was adequately tested through robustness testing using the instrumental variable method and propensity score matching method.

#### 3.2.3. Mediation Effect Test Results

Theoretical analyses revealed the positive effects of physical exercise on adolescents’ school adjustment, further leading to the potential mechanism of action examined in this study: whether negative emotions and prosocial behavior play a mediating role in the aforementioned causal relationship. This study was conducted based on the mediation test proposed by Baron et al. (Baron & Kenny, 1986). Table 5 demonstrates the results of the test for mediating effects. Columns (1) and (2) show the results of the test for the mediating variable negative emotions. The results in column (1) indicate that physical exercise significantly reduces negative emotions, whereas in column (2), the inclusion of the independent and mediating variables resulted in a significant inhibitory effect of negative emotions on school adjustment, and the effect of physical exercise on school adjustment remained significant. This suggests that negative emotions play a partial mediating role in the mechanism by which physical exercise affects school adjustment, i.e., physical exercise improves adolescents’ school adjustment by alleviating negative emotions.

Similarly, Columns (3) and (4) test the mediating role of prosocial behavior. The findings in Column (3) indicate that physical exercise significantly enhances prosocial behavior, while the results in Column (4) show that after considering both prosocial behavior and physical exercise, prosocial behavior significantly enhances adolescents’ school adjustment, and the effect of physical exercise remains significant. This suggests that prosocial behavior also plays a partial mediating role in the mechanism by which physical exercise affects school adjustment, i.e., physical exercise indirectly contributes to adolescents’ school adjustment by enhancing prosocial behavior. In the analysis of Column (5), the effects of both negative emotions and prosocial behavior were supported by the simultaneous inclusion of these two mediating variables, further confirming the existence of the mediating effect.

The mediating effects of negative emotions and prosocial behavior were further decomposed by the KHB method, and the results in Table 6 showed that the direct effect coefficient between physical exercise on school adjustment was 0.216, and the mediating effect coefficients of negative emotions and prosocial behavior were 0.024 and 0.117, which accounted for 6.73% and 32.77% of the total effect, respectively. Taken together, the results suggest that physical exercise promotes adolescents’ school adjustment through both reducing negative emotions and enhancing prosocial behavior. Based on this, hypotheses 2 and 3 of this study were also validated.

## 4. Discussion

Using the most recent and nationally representative data from the China Education Panel Survey, this study explored the effects of physical exercise on adolescents’ school adjustment, taking into account endogeneity issues and sample selection bias. There are two main findings in the study: on the one hand, after robustness analyses through the instrumental variables method and propensity score matching method, the regression results both indicate that the positive effect of physical exercise on adolescents’ school adjustment is significant. On the other hand, after further testing for mediating effects, it was found that negative emotions and pro-social behaviors played a mediating role in the pathway of physical exercise for adolescents’ school adjustment.

### 4.1. The Direct Impact of Physical Exercise on Adolescents’ School Adjustment

Improving and developing adolescents’ school adaptive skills is crucial for their future socialization growth, personality improvement, etc., and better school adaptive skills can contribute to adolescents’ personality development and value formation, while laying the foundation for lifelong development (Suhlmann et al., 2018). The results of this study validate the Developmental Task Theory, which suggests that participation in physical exercise or other recreational activities allows individuals to accomplish some of the “developmental tasks” of the current stage, thereby enhancing their adaptive capacity. Developmental tasks in adolescence include building relationships with others, developing the knowledge, skills, and attitudes needed by individuals, and developing socially responsible behaviors (Manning, 2002). In conjunction with the Integrated Developmental Task Theory of Adaptive Development, physical exercise promotes adolescent school adjustment for the following possible reasons: first, physical exercise develops skills and socially responsible behaviors in adolescents, and in physical exercise, students must abide by the rules and discipline of the game. This rule-following behavior helps students develop a good sense of discipline in the school environment and comply with school rules and regulations. In turn, the development of a sense of rules helps students to better adapt to school life and observe classroom discipline, which in turn improves learning efficiency and academic performance; second, physical exercise develops good attitudes needed by adolescents. Physical exercise emphasizes the courage to fight and not to be proud of victory or defeat, and this spirit motivates students to be able to cope positively, strive hard and keep improving when facing academic challenges. Whether in physical exercise or in academics, this enterprising attitude can help students maintain a positive attitude toward learning, overcome difficulties, and improve academic performance (Yan et al., 2020); finally, physical exercise develops good interpersonal relationships with others at the adolescent stage. During physical exercise, students need to learn how to collaborate, communicate and cooperate with others. The sense of cooperation developed through physical exercise can help students work better with peers in classroom group activities and school socialization, enhance collective cohesion and teamwork, and improve school adjustment.

### 4.2. The Indirect Effects of Physical Exercise on Adolescents’ School Adjustment

The indirect effects of physical exercise on adolescents’ school adaptation operate through two primary mediating pathways, which can be effectively framed within Havighurst’s developmental tasks theory. This theory posits that adolescence is a critical period for mastering key tasks, including emotional maturity and developing responsible social behavior. Our findings illustrate how physical exercise facilitates the successful navigation of these very tasks.

Firstly, the mediating role of negative emotions. According to Fredrickson’s broaden-and-build theory, especially the undoing hypothesis, positive emotional experiences can counteract the adverse consequences of negative affect, including narrowed repertoires of thought and action, heightened physiological arousal, and persistent stress (Fredrickson, 2001). Havighurst identified emotional development as a paramount task for adolescents. Physical exercise serves as a powerful engine for this development. Physical exercise-induced physiological responses, such as the release of β-endorphin, reduce the levels of stress hormones (for example, adrenaline and cortisol) and generate pleasure and a sense of achievement (X. Wang et al., 2024). These brief positive states can interrupt cycles related to anxiety or depression, and help adolescents recover from academic or interpersonal stress, thereby providing the psychological space and resilience needed for better school adaptation. Moreover, the rich social interactions inherent in physical activity, including peer encouragement and shared practice experiences, foster positive socioemotional bonds (Eather et al., 2023). The resulting sense of belonging, enjoyment, and friendship directly counter loneliness and alienation. In line with broaden-and-build theory, the cognitive broadening associated with positive emotions increases adolescents’ willingness to explore, flexibility in managing peer relationships, and constructive approaches to academic challenges, which in turn enhances school adjustment (Azpiazu et al., 2024). In sum, physical exercise may affect the negative emotion pathway through a dual route: it reduces negative affect through physiological and social processes, and it generates positive emotions that undo residual negative affect and build more adaptive psychological resources.

Second, physical exercise promotes adolescents’ school adjustment by enhancing prosocial behavior, with the mediating effect of prosocial behavior accounting for 32.77% of the total effect. This underscores its role as a crucial mechanism and directly aligns with the key Havighurst task of developing socially responsible behavior. Social learning theory provides a foundational mechanism for this developmental pathway. This theory posits that individuals acquire new behaviors through observational learning and the imitation of modeled attitudes, and emotional responses (Coşkun & Ünal, 2024). The context of team-based physical activities (e.g., basketball and soccer) serves as a powerful social learning environment. When adolescents observe peers demonstrating rule compliance, fair play, teamwork, and selflessness during exercise, they are more likely to imitate and internalize these prosocial conducts (Hui et al., 2022). Empirical evidence corroborates this: for instance, a structured 12-week basketball intervention program was shown to significantly enhance students’ sense of social responsibility and prosocial behaviors, demonstrating how a designed sport setting can effectively foster these qualities through modeling and practice (Xie et al., 2025). In this process, adolescents are not passive imitators; they actively practice and adjust their social behaviors based on perceived consequences. Through this active engagement, social norms such as fairness and cooperation are internalized into stable prosocial dispositions. The generalization of these learned behaviors is evident in findings that adolescents who actively participate in physical exercise exhibit more prosocial behavior and less aggression, as well as demonstrate better cooperation and problem-solving skills when facing challenges in broader contexts (Iglesias Gallego et al., 2020). When these internalized prosocial patterns are transferred to wider school settings—including classroom interactions, group assignments, and dormitory life—they foster more harmonious peer relations, more positive learning attitudes, and fewer interpersonal conflicts, thereby concretely promoting school adjustment. Therefore, physical exercise is not merely bodily training; it is a dynamic and immersive social learning environment where prosocial competencies are acquired, practiced, and generalized, ultimately facilitating successful school adjustment.

### 4.3. Recommendation

It is recommended that schools should recognize the developmental tasks characteristic of adolescence, foster students’ motivation to engage in physical exercise, and leverage existing policy frameworks to fully implement Physical Education (PE) and Health courses. Schools should also provide safe, high-quality, and diverse sports facilities and ensure sufficient in-school time for daily physical activity, enabling students to accomplish stage-specific developmental tasks through exercise and thereby better adjusting to school life. Second, greater attention should be paid to adolescents’ emotional problems. Teachers and parents should jointly enhance attention to adolescents’ emotional and mental states, cultivate caring and understanding interpersonal climates in daily settings, and support the maintenance of positive affect. Schools should strengthen mental-health counseling infrastructure and use standardized instruments to screen for mental health concerns, allowing for timely identification and intervention. Third, adolescents’ prosocial behavior should be actively cultivated. Given its central role in education and healthy development, schools and families should adopt targeted interventions, such as possible-selves exercises (imagining how to become a better self), reflecting on one’s “best possible self,” and viewing brief videos that model prosocial behavior to nurture and strengthen prosocial tendencies, thereby improving school adjustment and laying a stronger foundation for lifelong development.

### 4.4. Limitations

Although this study explored the effects and the underlying mechanisms between physical exercise and school adjustment in adolescents and achieved the positive findings described above, there are some shortcomings in this study. First, the China Education Panel Survey 2014–2015 data used in this study is a national tracking survey data that has been widely used, covering multiple dimensions of student development. However, the investigation of adolescents’ physical exercise is not detailed enough to assess the extent of adolescents’ physical exercise only in terms of time, and it is not possible to analyze the impact of physical exercise intensity and programs on adolescents’ school adjustment. Second, this study discussed adolescents’ school adjustment, but the China Education Panel Survey data came from middle school students, and the sample did not yet include students at other educational stages, such as elementary school students or high school students, so the general applicability of the findings needs to be further verified. Third, regarding the underlying mechanisms, this study examined negative emotion and prosocial behavior as parallel mediators. While this approach clearly demonstrates their independent contributions, it potentially oversimplifies their interrelationship. A more complex model, such as a serial mediation model where physical exercise reduces negative emotion, which in turn facilitates prosocial behavior to promote adjustment, is theoretically plausible. However, the relationship between negative emotion and prosocial behavior is ambiguous in the literature, with evidence showing both inhibitory and facilitative effects (Van Kleef & Lelieveld, 2022; S. Wang et al., 2025). Therefore, we prioritized testing the robust independent pathways. Future research should explicitly test such sequential pathways to provide a more nuanced understanding of the psychological and behavioral processes involved.

## 5. Conclusions

Using data from the China Education Panel Survey 2014–2015, this study empirically analyzed the effects of physical exercise on adolescents’ school adaptation and its mechanism of action using the Developmental Task Theory as a framework, enriching the findings of the study on physical exercise and school adaptation: (1) Physical exercise significantly enhances adolescents’ school adaptation ability. After considering potential endogeneity issues such as reverse causation, omitted variables, and measurement error, the findings still hold after a series of robustness tests using instrumental variables method and propensity score matching method. (2) The mediation effect analysis showed that there was a significant mediation effect between negative emotions and pro-social behaviors, and that adolescents’ participation in physical exercise could significantly reduce their negative emotions and promote pro-social behaviors, which in turn would have an impact on adolescents’ school adjustment.

## Figures and Tables

**Table 1:** Results of Descriptive Statistics.

Variable	Sample Size	Mean	SD	Minimum	Maximum
School Adjustment	8707	26.60	4.519	9	36
Negative Emotions	8707	19.63	7.602	9	45
Prosocial Behavior	8707	11.41	2.275	3	15
Physical Exercise	8707	2.526	1.522	−4.605	5.886
Gender	8558	0.518	0.500	0	1
Only child	8597	0.448	0.497	0	1
Own health status	8647	3.869	0.933	1	5
Health education status	8591	0.629	0.483	0	1
Obesity perception	8650	0.376	0.484	0	1
Live in school	8546	0.299	0.458	0	1
Household Economic Status	8377	2.820	0.606	1	5
Father’s education	8385	10.44	3.154	0	19
Mother’s education	8354	9.858	3.473	0	19
Father–child relationship	8675	2.506	0.579	1	3
Mother–child relationship	8670	2.705	0.501	1	3
Parental relationships	8622	0.899	0.302	0	1
Type of school	8707	0.938	0.242	0	1
Ranking of the school	8707	3.984	0.839	1	5
Location of the school	8520	2.340	0.790	1	3
School physical facilities	8707	0.876	0.330	0	1

**Table 2:** Regression Model of Physical Exercise effecting Adolescents’ School Adjustment.

Variable	(1)	(2)	(3)	(4)
Physical Exercise	0.616 ***	0.462 ***	0.350 ***	0.357 ***
	(0.031)	(0.033)	(0.033)	(0.034)
Gender		−1.428 ***	−1.196 ***	−1.160 ***
		(0.092)	(0.091)	(0.092)
Only child		1.196 ***	0.500 ***	0.436 ***
		(0.097)	(0.104)	(0.107)
Own health status		1.207 ***	0.898 ***	0.897 ***
		(0.052)	(0.053)	(0.054)
Health education status		0.944 ***	0.819 ***	0.768 ***
		(0.096)	(0.094)	(0.095)
Obesity perception		−0.430 ***	−0.435 ***	−0.471 ***
		(0.096)	(0.093)	(0.095)
Live in school		−0.839 ***	−0.661 ***	−0.491 ***
		(0.106)	(0.109)	(0.120)
Household Economic Status			0.501 ***	0.474 ***
			(0.083)	(0.084)
Father’s education			0.093 ***	0.088 ***
			(0.020)	(0.020)
Mother’s education			0.106 ***	0.099 ***
			(0.018)	(0.018)
Father–child relationship			0.932 ***	0.953 ***
			(0.093)	(0.094)
Mother–child relationship			1.263 ***	1.246 ***
			(0.106)	(0.107)
Parental relationships			0.778 ***	0.776 ***
			(0.173)	(0.174)
Type of school				0.768 ***
				(0.212)
Ranking of the school				0.095
				(0.061)
Location of the school				0.137
				(0.074)
School physical facilities				−0.193
				(0.131)
Constant	25.044 ***	20.820 ***	12.681 ***	11.597 ***
	(0.092)	(0.236)	(0.391)	(0.498)
Observations	8707.000	8107.000	7539.000	7380.000
R^2^	0.043	0.176	0.254	0.257

Note: (1) *** *p* < 0.001; (2) Robust standard errors in parentheses. The same as below.

**Table 3:** Physical Exercise and Adolescent School Adjustment: IV-2SLS Estimates.

Variable	(1)	(2)	(3)	(4)
Panel B	Results of phase II			
Physical Exercise	2.858 ***	1.954 ***	1.348 ***	1.354 ***
	(0.158)	(0.153)	(0.159)	(0.162)
Panel A	Results of phase I			
School-level sports participation rates (other than individual)	0.854 ***	0.806 ***	0.750 ***	0.752 ***
	(0.036)	(0.119)	(0.041)	(0.042)
Individual characteristics		Control	Control	Control
Family characteristics			Control	Control
School characteristics				Control
Constant	0.369 ***	−0.340 **	−0.919 ***	−0.891 ***
	(0.091)	(0.118)	(0.160)	(0.242)
Observations	8707	8107	7539	7380
Minimum eigenstatistic value	577.48	101.61	53.13	41.34

Note: ** *p* < 0.01, *** *p* < 0.001.

**Table 4:** Effects of Physical Activity on Adolescents’ School Adjustment: PSM Estimation Results.

Matching Method	T	C	ATT	SE
Nearest neighbor matching	27.634	26.596	1.037	0.120
Radius matching	27.634	26.590	1.044	0.109
Kernel matching	27.624	26.579	1.055	0.109

**Table 5:** Results of the Mediation Effect Test.

Variable	(1)	(2)	(3)	(4)	(5)
	NE	SA	PB	SA	SA
Physical exercise	−0.169 **	0.331 ***	0.243 ***	0.233 ***	0.216 ***
	(0.057)	(0.030)	(0.017)	(0.030)	(0.030)
Negative emotions		−0.152 ***			−0.143 ***
		(0.006)			(0.006)
Prosocial behavior				0.509 ***	0.480 ***
				(0.021)	(0.020)
Individual characteristics	Control	Control	Control	Control	Control
Family characteristics	Control	Control	Control	Control	Control
School characteristics	Control	Control	Control	Control	Control
Constant	35.476	17.610	6.673	8.834	14.099
	(0.919)	(0.536)	(0.273)	(0.507)	(0.538)
Observations	7023	7023	7023	7023	7023
R^2^	0.123	0.312	0.126	0.312	0.362

Note: ** *p* < 0.01, *** *p* < 0.001.

**Table 6:** Intermediation Model Effect Test.

	Coefficient	SE	Percentage of Relative Effects
Total effect	0.357	0.029	100%
Direct effect	0.216	0.030	60.50%
Total indirect effect	0.141	0.013	39.50%
Indirect effect 1 (Negative emotions)	0.024	0.008	6.73%
Indirect effect 2 (Prosocial behavior)	0.117	0.010	32.77%

## Data Availability

The data utilized in this study are sourced from the National Survey Research Center (NSRC) at Renmin University of China. Due to licensing restrictions, these data are not publicly accessible. The original CEPS datasets can be accessed by registered users who complete the application process via the official website: http://ceps.ruc.edu.cn/. Additionally, the Stata do-file used for data processing is provided as Supplementary Materials.

## Supplementary Materials

The following supporting information can be downloaded at: https://www.mdpi.com/article/10.3390/bs15121602/s1.
