# Supplementary material for: The Effects of Physical Exercise on Adolescents’ School Adjustment and Path Analysis—Evidence from the China Education Panel Survey
Source: Behav Sci (Basel). 2025 Nov 21;15(12):1602. doi: 10.3390/bs15121602 (PMC12729406; doi:10.3390/bs15121602)
Supplement: Supplementary file 1 [file behavsci-15-01602-s001.zip › behavsci-3947381-supplementary.pdf]

```
use "C:\Users\Chw\Desktop\CEPS\2014-2015Chinese\cepsw2studentCN.dta" //2014-2015 数据

//How to convert garbled characters in a database?
//clear
//cd"C:\Users\Chw\Desktop\CEPS\student2014-2015data"
//unicode encoding set gb18030
//unicode analyze cepsw2studentCN.dta
//unicode translate cepsw2studentCN.dta
//unicode translate cepsw2studentCN.dta， invalid

//First, merge the data in the following order: student data, parent data, and principal data.

//This demonstration primarily uses CEPS 2014-2015 data. We use data from successfully followed-up students and newly enrolled students, excluding those who were not followed up.
gen zfqk=w2status //First, create a follow-up status variable.
tabu zfqk,missing //Let's see how many students were not followed up.
drop    if zfqk==2    //Delete those who were not successfully followed up.




gen dlts=w2c13a //Define new exercise days
gen dlsj=w2c13b //Define new exercise time
drop if dlsj>360// Remove extreme cases where daily exercise time exceeds 360 minutes.
drop if dlts==. //Remove the number of people with missing exercise days.
gen mtdlsc=(dlts*dlsj/7)+0.01 //Calculate the average daily exercise duration for each individual, and add 0.01 to all individuals for easier calculation of the natural logarithm later.
gen lnmtdlsc=ln(mtdlsc) //Take the natural logarithm

//Cleaning prosocial behavior
drop if w2d0101==.      //Remove missing values from the question items.
drop if w2d0102==.
drop if w2d0103==.
gen qshxw=w2d0101+w2d0102+w2d0103


//School adjustment can be categorized into academic adjustment, interpersonal adjustment, and behavioral adjustment.
tabu w2b02,missing //Examine the missing values of the academic adaptation items.
tabu w2b03,missing
tabu w2b04,missing
drop if w2b02==.      //Remove missing values from the question items.
drop if w2b03==.
drop if w2b04==.
```

```
gen xysy=w2b02+ w2b03 + w2b04 //Add up the items from the academic adaptation test to get the academic adaptation score.

tabu w2b0605,missing
tabu w2b0606,missing
tabu w2b0608,missing //Examine the missing values for the interpersonal adaptation items.
drop if w2b0605==.
drop if w2b0606==.
drop if w2b0608==.      //Remove missing values from the question items.
gen rjsy=w2b0605 + w2b0606 + w2b0608   //Add up the items related to interpersonal adaptation to obtain the interpersonal adaptation score.

tabu w2b0607,missing
tabu w2b0609,missing
tabu w2b0610,missing //Examine the missing values of the behavioral adaptation items.
drop if w2b0607==.    //Remove missing values from the question items.
drop if w2b0609==.
drop if w2b0610==.
recode w2b0609(1=4)(2=3)(3=2)(4=1)
recode w2b0610(1=4)(2=3)(3=2)(4=1)
gen xwsy=w2b0607+w2b0609+w2b0610     //The behavioral adaptation score is obtained by adding up the items related to behavioral adaptation.

gen xxsy=xysy+rjsy+xwsy //The school adaptation score is obtained by adding up all dimensions.

//Cleaning negative emotions
drop   if w2c2501==.
drop   if w2c2502==.
drop   if w2c2503==.
drop   if w2c2504==.
drop   if w2c2505==.
drop   if w2c2506==.
drop   if w2c2507==.
drop   if w2c2508==.
drop   if w2c2509==.
gen fxqx1=w2c2501
recode fxqx1(1=5)(2=4)(3=3)(4=2)(5=1) //Reverse scoring of negative emotions
gen fxqx2=w2c2502
recode fxqx2(1=5)(2=4)(3=3)(4=2)(5=1)
gen fxqx3=w2c2503
recode fxqx3(1=5)(2=4)(3=3)(4=2)(5=1)
gen fxqx4=w2c2504
recode fxqx4(1=5)(2=4)(3=3)(4=2)(5=1)
gen fxqx5=w2c2505
```

```
recode fxqx5(1=5)(2=4)(3=3)(4=2)(5=1)
gen fxqx6=w2c2506
recode fxqx6(1=5)(2=4)(3=3)(4=2)(5=1)
gen fxqx7=w2c2507
recode fxqx7(1=5)(2=4)(3=3)(4=2)(5=1)
gen fxqx8=w2c2508
recode fxqx8(1=5)(2=4)(3=3)(4=2)(5=1)
gen fxqx9=w2c2509
recode fxqx9(1=5)(2=4)(3=3)(4=2)(5=1)
gen    fxqxT=fxqx1+fxqx2+fxqx3+fxqx4+fxqx5+fxqx6+fxqx7+fxqx8+fxqx9 //Add up all the answers to get the total negative emotion score.
gen
fxqxZ=w2c2501+w2c2502+w2c2503+w2c2504+w2c2505+w2c2506+w2c2507+w2c2508+w2c250
9



/
//Cleaning personal condition
//gender
gen xb=a01
drop if xb==.
recode xb(2=0)    //Define girls as 0 and boys as 1.

//Cleaning up only children
gen dszn=w2a05
drop if dszn==. //Remove missing values for only child
recode    dszn(2=0)

//Cleansing health
gen jkzk=w2c04
drop if jkzk==.

//Cleaning and health education situation
gen jkjyk=w2c21
drop if jkjyk==.
recode    jkjyk(2=0)    //Those who have never attended a health education class are defined as 0, and those who have are defined as 1.

//Cleanse the senses of obesity
gen    scps=w2c03
drop if scps==.
recode    scps(1=0)(2=0)(3=0)(4=1)(5=1)    //Options 1, 2, and 3 are defined as "not fat" (0), and options 4 and 5 are defined as "fat" (1).
```

```
//Cleaning; whether overnight stay is required.
gen xxls=w2b15
recode    xxls(2=0)
drop if xxls==.


//Cleaning household variables
//Economic situation
gen jtjjqk=w2be23
drop    if jtjjqk==.

//Parents' education level
gen fqxl=w2be08
recode fqxl(1=0)(2=6)(3=9)(4=11)(5=11)(6=12)(7=15)(8=16)(9=19)
gen mqxl=w2be17
recode mqxl(1=0)(2=6)(3=9)(4=11)(5=11)(6=12)(7=15)(8=16)(9=19)

//Father-son relationship
gen fzgx=w2a22 //1 = Not close 3 = Very close
//Mother-child relationship
gen mzgx=w2a23    //1 = Not close 3 = Very close
//Parental relationship
gen fmgx=w2a17
recode fmgx(2=0) //1 = Good 0 = Bad


//Cleaning school-level variables
//School nature
gen xxxz=w2pla01
recode xxxz(1=1)(2=0)(3=0)(4=0) //Public schools are 1, others are 0.

//School Rankings
gen xxpm=w2pla03 //1 = worst 5 = best


//School Location
gen xxqw=w2pla19
recode xxqw(1=3)(2=3)(3=2)(4=2)(5=1)


//School sports facilities
gen tycc=w2pla06
recode tycc(2=0)
```

//Export the data as a Word document:
//ssc install logout
logout, save(mytable) word replace: tabstat xxsy qshxw fxqxZ lnmtdlsc xb dszn jkzk jkjyk scps xxls jtjjqk fqxl mqxl fzgx mzgx fmgx xxxz xxpm xxqw tycc ///
, s(N mean p50 sd min max) f(%12.3f) c(s)

//OLS regression results
reg xxsy lnmtdlsc
est store m1
reg xxsy lnmtdlsc xb dszn jkzk jkjyk scps xxls ,r
est store m2
reg xxsy lnmtdlsc xb dszn jkzk jkjyk scps xxls jtjjqk fqxl mqxl fzgx mzgx fmgx, r
est store m3
reg xxsy lnmtdlsc xb dszn jkzk jkjyk scps xxls jtjjqk fqxl mqxl fzgx mzgx fmgx xxxz xxpm xxqw tycc , r
est store m4
esttab m1 m2 m3 m4 using C:\Users\Chw\Desktop\CEPS\regoutOLS.rtf, b(%12.3f) se(%12.3f) nogap compress s(N r2 ar2) star(* 0.05 ** 0.01 *** 0.001)

estat imtest, white //Heteroscedasticity test: If heteroscedasticity exists, the results should be output using OLS with robust standard errors.
reg xxsy lnmtdlsc xb dszn jkzk jkjyk scps xxls jtjjqk fqxl mqxl fzgx mzgx fmgx xxxz xxpm xxqw tycc blxw , r //OLS+ Robust Standard Error

//Instrumental variable method
bys schids: egen mean=mean(lnmtdlsc) // The physical exercise levels of students in each school are treated as a group, and the overall physical activity level of the school, excluding the individual who answered the questionnaire, is calculated. This can be achieved using STATA software: for example,
by schids: egen ct = count(lnmtdlsc) // bys group: egen mean=mean(x) by group: egen ct = count(x) gen meani = (mean*ct-x)/(ct-1)
gen meani = (mean*ct-lnmtdlsc)/(ct-1) // Where group is the group you belong to, mean is the average value, x is the variable you are interested in, and count is the count. The final meani is the mean value of the group excluding individual i.
ivregress 2sls xxsy (lnmtdlsc = meani), first
ivregress 2sls xxsy xb dszn jkzk jkjyk scps xxls (lnmtdlsc = meani), first
ivregress 2sls xxsy xb dszn jkzk jkjyk scps xxls jtjjqk fqxl mqxl fzgx mzgx fmgx (lnmtdlsc = meani), first
ivregress 2sls xxsy xb dszn jkzk jkjyk scps xxls jtjjqk fqxl mqxl fzgx mzgx fmgx xxxz xxpm xxqw tycc (lnmtdlsc = meani), first // Two-stage least squares method, reporting the first-stage

regression (r, first).

estat firststage // Check if the F-value is greater than the value of the 2SLS with a deviation of 10. If it is greater than and > 10, it indicates that the instrumental variable is a strong instrumental variable.
estat overid
estat endog // The Durbin-Wu-Hausman endogeneity test indicates that endogeneity exists if the p-value is significant, and using the instrumental variable method is the correct approach.

//PSM
gen psmty = 0
replace psmty= 1 if w2c13a>=3 & w2c13b >=30
list psmty w2c13a w2c13b in 1/20
psmatch2 psmty xb dszn jkzk jkjyk xxls jtjjqk fqxl mqxl fzgx mzgx fmgx xxxz xxpm xxqw tycc , outcome(xxsy) logit ate ties qui //PSM matching based on logit
pstest, both graph //Test whether the matched data are balanced

//Instructions for nearest neighbor matching, caliper matching, radius matching, and kernel matching in propensity score matching.
//Nearest neighbor matching: psmatch2 t x1 x2,outcome(y) n(1)——1-to-1 matching; psmatch2 t x1 x2,outcome(y) n(k)——1-to-k matching ///
https://blog.csdn.net/celine0227/article/details/121052941 1:4 matching evidence
psmatch2 psmty xb dszn jkzk jkjyk xxls jtjjqk fqxl mqxl fzgx mzgx fmgx xxxz xxpm xxqw tycc , outcome(xxsy) logit ate ties common n(4) qui
set seed 10101
bootstrap r(att)r(atu)r(ate), reps(500):psmatch2 psmty xb dszn jkzk jkjyk xxls jtjjqk fqxl mqxl fzgx mzgx fmgx xxxz xxpm xxqw tycc , outcome(xxsy) logit ate ties common n(4)
//Nearest neighbor matching within calipers: psmatch2 t x1 x2,outcome(y) n(k) caliper(real) where real is replaced with the number of calipers. The typical caliper value is 0.1.
sum _pscore calculate the standard deviation of pscore, then multiply it by 0.25, resulting in 0.035. This means that the range of the caliper is approximately equal to0.03
psmatch2 psmty xb dszn jkzk jkjyk xxls jtjjqk fqxl mqxl fzgx mzgx fmgx xxxz xxpm xxqw tycc , outcome(xxsy) logit ate ties common n(4) cal(0.03) qui
set seed 10101
bootstrap r(att)r(atu)r(ate), reps(500):psmatch2 psmty xb dszn jkzk jkjyk xxls jtjjqk fqxl mqxl fzgx mzgx fmgx xxxz xxpm xxqw tycc , outcome(xxsy) logit ate ties common n(4) cal(0.03) qui
//Radius matching: psmatch2 t x1 x2,outcome(y) radius caliper(real) real is replaced with the number of calipers
psmatch2 psmty xb dszn jkzk jkjyk xxls jtjjqk fqxl mqxl fzgx mzgx fmgx xxxz xxpm xxqw tycc , outcome(xxsy) logit ate ties common radius cal(0.03) qui
set seed 10101
bootstrap r(att)r(atu)r(ate), reps(500):psmatch2 psmty xb dszn jkzk jkjyk xxls jtjjqk fqxl mqxl fzgx

mzgx fmgx xxxz xxpm xxqw tycc , outcome(xxsy) logit ate ties common radius cal(0.03) qui
//Kernel matching: `psmatch2 t x1 x2, outcome(y) kernel kerneltype()` `kerneltype: normal biweight epan uniform tricube` Kernel matching defaults to `epan`. Default bandwidth is 0.06.
psmatch2 psmty xb dszn jkzk jkjyk xxls jtjjqk fqxl mqxl fzgx mzgx fmgx xxxz xxpm xxqw tycc , outcome(xxsy) kernel logit ate ties common qui
set seed 10101
bootstrap r(att)r(atu)r(ate), reps(500):psmatch2 psmty xb dszn jkzk jkjyk xxls jtjjqk fqxl mqxl fzgx mzgx fmgx xxxz xxpm xxqw tycc , outcome(xxsy) kernel logit ate ties common qui

//Kernel density function plot
//Before matching
twoway(kdensity _pscore if _treat==1 , legend(label(1 "Treat"))) ///
(kdensity _pscore if _treat==0 , legend(label(2 "Control"))), ///
xtitle(Pscore>) title("Before Matching")
graph save a1,replace
//After matching
twoway(kdensity _pscore if _treat==1 , legend(label(1 "Treat"))) ///
(kdensity _pscore if _weight!=1&_wei!=., legend(label(2 "Control"))), ///
xtitle(Pscore) title("After Matching")
graph save a2,replace
graph combine a1.gph a2.gph,scheme(burd)

//If the result report is too long and requires line breaks, simply run the command `set linesize 255` and then run another one to resolve the issue.
//How do intermediaries operate? Stepwise regression is needed.

reg fxqxZ lnmtdlsc xb dszn jkzk jkjyk xxls jtjjqk fqxl mqxl fzgx mzgx fmgx xxxz xxpm xxqw ssb
reg xxsy fxqxZ lnmtdlsc xb dszn jkzk jkjyk xxls jtjjqk fqxl mqxl fzgx mzgx fmgx xxxz xxpm xxqw ssb
reg qshxw lnmtdlsc xb dszn jkzk jkjyk xxls jtjjqk fqxl mqxl fzgx mzgx fmgx xxxz xxpm xxqw ssb
reg xxsy qshxw lnmtdlsc xb dszn jkzk jkjyk xxls jtjjqk fqxl mqxl fzgx mzgx fmgx xxxz xxpm xxqw ssb
reg xxsy fxqxZ qshxw lnmtdlsc xb dszn jkzk jkjyk xxls jtjjqk fqxl mqxl fzgx mzgx fmgx xxxz xxpm xxqw ssb

//This is the Sobel test:sgmediation xxsy, mv(zwkz fxqxT) iv(lnmtdlsc1) cv(xb dszn jkzk jkjyk xxls jtjjqk fqxl mqxl fzgx mzgx fmgx xxxz xxpm xxqw ssb)

//KHB Intermediary Method
khb regress xxsy lnmtdlsc || fxqxZ qshxw , concomitant(xb dszn jkzk jkjyk xxls jtjjqk fqxl mqxl

fzgx mzgx fmgx xxxz xxpm xxqw ssb)
khb regress xxsy lnmtdlsc || fxqxZ qshxw , concomitant(xb dszn jkzk jkjyk xxls jtjjqk fqxl mqxl fzgx mzgx fmgx xxxz xxpm xxqw ssb) summary disentangle notable
